# Swansea Hospital

**Published:** 1891-07-11

**Authors:** 


### THE HOSPITAL CLINIC.

### SWANSEA HOSPITAL.

Gangrene of Hand and Arm in a Woman aged 72 years; Amputation immediately below Shoulder-joint, Abdominal Abscess Recovery. Under the care of Mr. H. A. Latimer, Surgeon to the Swansea Hospital.

On the evening of September 10th, 1890, S. C., aged 72, was admitted into Swansea Hospital. A fortnight before admission she pricked a finger of the left hand when picking blackberries, and the wound became inflamed. On the 6th of the month she called in a medical man and was under his treatment until the 10th, when she was advised to enter the hospital. On admission there the whole hand was gangrenous, and the tendons on the dorsal surface were exposed; the forearm was swollen, red, and crepitating on pressure—evident sphacelus—and a red blush extended up to the axilla. As the skin was fairly free from mischief at that part, Mr. Latimir amputated, by the circular method, at a point about three inches below the shoulder-joint. A drainage tube was inserted, and the wound was dressed with salalembroth wool, after its surface had been dusted with iodoform and boracic acid powder. The patient was very weak and exhausted by illness and was ordered brandy and egg mixture every third hour.

Sept. 12th.—Removed drainage tube; as it was offensive the wound was dressed by thoroughly syringing out stump with iodine water; the skin all round it was painted with tincture of iodine, and, after replacing the drainage tube, dressings of sublimated wood wool were applied.

Sept. 13th.—Again dressed wound, which was looking healthy. Syringed stump and painted with iodine tincture. A quantity of discharge had drained away, but this discharge was free from smell. Left out drainage tube. Re-dressed with sublimated wool. Is taking nourishment well, and sleeps fairly.

Sept. 20th.—Now out of the hospital. Same dressings have been applied as described above. Three-fourths of the wound surface has now healed, and the whole is perfectly healthy and without blush.

Sept. 30th.—Have been dressing with terebene and linseed oil for past week, and the stump has all but healed. She is very weak.

Oct. 4th.—Although the stump has nearly healed the patient has been getting steadily weaker. About the 20th ult. she called attention to a swelling in the abdomen; it was situated below the liver (clearly not connected with that organ), was freely movable, gave very little pain on handling, and was not attached to the abdominal wall or to any organ beneath it. There being a history of chronic constipation, an enema was administered with the long tube, but, although the bowels acted well after this was done, no diminution in the size of the swelling occurred. There was no rise of temperature. She complained constantly of the swelling, but no constitutional trouble seemed to follow it until, on the 4th of October, she suddenly became feverish and delirious. A consultation was then held with Dr. T. D. Griffiths, of Swansea, who agreed that the swelling was due to an abscess situated within the abdomen, behind the abdominal wall and in front of the peritoneum. She was urged to allow the abscess to be opened immediately, but absolutely refused her consent to this proceeding. But on October 5th Mr. Latimer, having obtained her consent, opened the abscess by making an incision of about 2½ inches in length, right through the abdominal wall, at a point just below and to the right of the umbelicus. A grooved needle was then plunged deeply into and along the tumour, and, this being used as a guide, the abscess was fully opened with a scalpel. Nearly a tumblerful of thick, laudable pus then came away. The cavity from which this came was then syringed out with solution of corrosive sublimate, and the abscess was dressed with salalembroth wool under a many-tailed bandage.

Oct. 6th.—Is much better.

Oct. 10th.—Has been dressed with antiseptic absorbent wools; no poultices. The thickening at the site of the abscess has almost disappeared.

Oct. 18th.—Is quite well, and goes out of doors again.

Note.—This case is interesting on account of the recovery from a very grave condition of disease of an old and feeble woman of 72 years of age. That one so advanced in years should have got well after a severe gangrenous process, an amputation, and an internal abscess of metastatic origin, is notable. The situation of the abscess calls for remark. It lay between the abdominal wall and the peritoneum. Had the patient persisted in her refusal to allow it to be opened, she would probably have died of peritonitis by its bursting within the abdomen and making its way through the peritoneum into the peritonial cavity. The improvement in her general condition which followed the evacuation of the matter was immediate and most marked.

